# Identifying the degree of genetic interactions using Restricted Boltzmann Machine—A study on colorectal cancer

**DOI:** 10.1049/syb2.12009

**Published:** 2020-12-08

**Authors:** Sujay Saha, Saikat Bandopadhyay, Anupam Ghosh

**Affiliations:** ^1^ Department of Computer Science & Engineering Heritage Institute of Technology Kolkata India; ^2^ Department of Computer Science & Engineering Netaji Subhash Engineering College Kolkata India

## Abstract

The phenomenon of two or more genes affecting the expression of each other in various ways in the development of a single character of an organism is known as gene interaction. Gene interaction not only applies to normal human traits but to the diseased samples as well. Thus, an analysis of gene interaction could help us to differentiate between the normal and the diseased samples or between the two/more phases any diseased samples. At the first stage of this work we have used restricted Boltzmann machine model to find such significant interactions present in normal and/or cancer samples of every gene pairs of 20 genes of colorectal cancer data set (GDS4382) along with the weight/degree of those interactions. Later on, we are looking for those interactions present in adenoma and/or carcinoma samples of the same 20 genes of colorectal cancer data set (GDS1777). The weight/degree of those interactions represents how strong/weak an interaction is. At the end we will create a gene regulatory network with the help of those interactions, where the regulatory genes are identified by using Naïve Bayes Classifier. Experimental results are validated biologically by comparing the interactions with NCBI databases.

## 1 INTRODUCTION

As defined by biologists, gene is a locus (or region) of DNA which is made up of nucleotides and is the molecular unit of heredity and the basis of the inheritance lies in transmission of genes to organism's offspring [1]. Gene forms the foundation of the central dogma of biology which consists of DNA replication, RNA transcription and protein translation. Experiments have proved that most of the characteristics of the living organisms are controlled by a collaboration of several different genes [1]. It is known that genes work together in a cell to make the cell function properly. There are some genes in the DNA known as the regulator gene, regulator or regulatory gene which controls one or more genes to increase or decrease the production of specific gene products (protein or RNA) thus modifying their gene expression patterns to activate developmental pathways, respond to environmental stimuli, or adapt to new food sources [2].

A group of functional relationships between a pair of genes is referred to as genetic interaction (GI). Bateson and Mendel (1909) [3] introduced one type of such relationships, called epistasis. Biological epistasis was then referred to as the effect of one allele masking the effect of another one [4]. After almost 9 years of this discovery, Fisher (1919) described statistical epistasis, originally called ‘epistacy’, which is a significant deviation of the phenotype of a double mutant from what is expected considering the phenotypes of the single mutants [5]. In literature, we have found so many statistical and computational methods that are used to detect and characterize those genes whose effects depends on other genes [6]. The main focus is on the genetic association studies of discrete and quantitative traits. The reason is most of the methods for detecting gene–gene interactions have been developed specifically for these study designs.

A gene regulatory network (GRN) is a collection of molecular regulators that interact with each other. These regulators can be DNA, RNA, proteins and so on. Many statistical and machine learning based methods have recently advanced in the construction of GRNs on some biological data sets [7]. All these methods had tried to identify the GRN by comparing the expression values among the genes of normal and diseased samples. The most common logic behind gene network inference is quite simple. When the expression level of a gene is perturbed and subsequently another gene's expression level is observed to change, then it can be inferred that the earlier gene is regulating the later one. Although the concept is simple, such measurements are very much complicated. The reason for this is most gene expression studies on diseased samples concern observational data [7].

Since identifying both the interaction as well as the strength of the interaction between every pair of genes in a GRN is our main goal thus we proposed an algorithm that will observe and measure the likelihood of the interaction between the genes of normal and diseased samples, as well as between the genes of various phases of diseased samples. Thus, the bonding between two individuals can be strongly determined through interactions only. More interactions an individual go through; more it learns about others and finds the best individual to interact with among others. This is very much similar with the working principle of restricted Boltzmann machine (RBM) model. That's why in this work we have used RBM model, where genes are allowed to interact with each other and by this we find the strength of the interaction, as well as the direction of the interaction between a pair of genes in that GRN using Naïve Bayes Classifier (NBC).

### 1.1 Restricted Boltzmann machine

Boltzmann machines (BMs) can be defined as bidirectionally connected stochastic neural network models [8]. A BM can be used to learn important aspects of an unknown probability distribution based on samples from this distribution. A RBM is a simplified version of BM where some restrictions are imposed on the network topology. Given some training data, learning a BM means adjust the BM parameters such that the probability distribution represented by the BM fits the training data as far as possible. Boltzmann machines consist of two types of units, so called visible and hidden neurons, which can be thought of as being arranged in two layers.

The visible units constitute the first layer and correspond to the components of an observation. The hidden units model dependencies between the components of observations.

**FIGURE 1:**
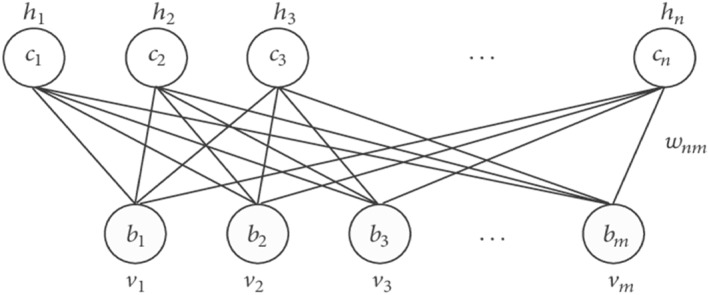
The undirected graph of an restricted Boltzmann machine with *n* hidden and *m* visible variables

The RBM, shown in Figure 1, is a bipartite undirected graph. It consists of m visible units (*v*_1_,…, *v*_*m*_) and n hidden units (*h*_1_, …, *h*_*n*_) to capture dependencies between observed variables (Fischer and Igel, 2012). In binary RBMs, the random variables (*V*, *H*) take values (*v*, *h*) ∈ {0, 1}^m+n^ and the joint probability distribution under the model is given by the following energy function:

(1) $$E\left(v,h\right)=-\sum_{i=1}^{m}\sum_{j=1}^{n}w_{ij}h_{i}v_{j}-\sum_{j=1}^{m}b_{j}v_{j}-\sum_{i=1}^{n}c_{i}h_{i}$$

For all *i* ∈ {1, …, *n*} and *j* ∈ {1, …, *m*}, *w*_*ij*_ is a real valued weight associated with the edge between units V_*j*_ and H_*i*_, whereas b_*j*_ and c_*i*_ are bias terms associated with the *j* ^th^ visible and the *i* ^th^ hidden variable, respectively. The graph of an RBM has only connections between the layer of hidden and visible variables but not between two variables of the same layer. In terms of probability this means that the hidden variables are independent given the state of the visible variables and vice versa [8].

## 2 RELATED EARLIER WORKS

In Genomics Study and computational biology, the discovery of gene connectivity/interaction networks from temporal expression data is one of the most pressing problems in computational biology. Researchers are working on the field of genomic data analysis and ranking genes which are both biologically and statistically significant based on a gene microarray experiment.

To start this research, we came across several works that have already been proposed to find the level of interactions between genes. Let's now briefly discuss those methods:

One such work, proposed in the study by Watkinson et al. [9], uses mutual information between the genes. Here, the relation between each pair of genes is used to build the synergy network. If the synergy level between gene pairs is high then that two genes shared a pathway and leads to a graphical representation of inferred gene–gene interactions associated with disease, in the form of a ‘synergy network’. The proposed approach is applied on a set of publicly available prostate cancer gene expression data sets and the results are also validated successfully.

Another new method, presented in [10], for estimating gene group interactions uses sparse canonical correlation analysis coupled with repeated random partition and sub‐sampling of the gene expression data set. This method infers these types of interactions using appropriate partial correlations between genes. The proposed approach is compared with several existing methods on simulated and real data sets. Experimental results show that the new procedure performs better than those earlier methods in terms of both the statistical measure as well as biological measure.

Another novel algorithm, titled as Signing of Regulatory Networks (SIREN), proposed in [11], can infer a regulatory type of interactions for each pair of connected gene of a GRN by computing a similarity score. SIREN score is estimated in four ways in this work, one by B‐Spline discretization of expression data, and another by calculating co‐occurrence scores for each combination of bins of the genes, third one is by rescaling of co‐occurrence scores, and the last one is by calculation of expected value of the rescaled co‐occurrence probability scores. The proposed approach is applied on three different benchmark GRNs, including *Escherichia coli*, prostate cancer, and an in silico constructed network. Experimental results show that the new method has approximately 68, 70, and 100 percent accuracy, for these networks, respectively.

Another work, based on differential co‐expression analysis, proposed in the study by Hsu et al. [12] is applied on *Saccharomyces Cerevisiae* to build differential co‐expression network. It identifies transcription factors that cause differential expressions under different situations. Result analysis found that differentially co‐expressed genes tend to participate in different pathways.

Correlation & entropy metrics based novel work is presented in the study by Seal et al. [13] to find the level of interaction between the genes applied on gene interaction networks. Experiments are done on three benchmark cancer data sets Colorectal, Leukaemia and CML. Results show some weighted graphs, where the weights along each edge represents the level of interaction between two genes in a particular network.

A two‐stage discovery‐confirmatory analysis is proposed in the study by Meng et al. [14] that explored potential gene–gene interactions for hypertension to take place. The first stage was an exhaustive pair wise search performed in 2320 early onset hypertensive cases with matched normotensive controls from the offspring cohort. In the second stage, identified gene–gene interactions were justified in an independent set of 694 subjects from the original cohort. Experimental results identified four unique gene–gene interactions susceptible to hypertension. Overall, this gene–gene interaction analysis helps to identify those genes which can provide more insights into the genetic background of blood pressure regulation.

A novel work is proposed by Saha et al. [15], which firstly uses three correlation measures, like Pearson, Spearman and Kendall‐Tau to find the interaction level in a gene interaction network. In the second phase of the experiment, entropy measure & Rough set theory are also used to determine the level of interaction between every pair of genes as well as finds the direction of interaction that indicates which gene regulates which other genes. Experiments are done on normal & diseased samples of colorectal cancer (CRC) data set (GDS4382) separately. Results are validated with NCBI database.

## 3 PROPOSED METHODOLOGY

In general, a GI between a pair of genes implies that the phenotype of a double mutant is different from what is expected from each individual mutant. Genome scale studies of quantitative GIs, in the last decade, were completed mainly using synthetic genetic array technology and RNA interference [6]. These studies raised many questions on the functional interpretation of GIs, like the relationship of genetic and molecular interaction networks, the usefulness of GI networks to infer gene function and co‐functionality, the evolutionary conservation of GI and so on [6]. Thus, gene expression (RNA expression) can be treated as an important parameter for constructing the GI. In this study, we have developed the gene–gene interaction from human colon expression data sets. Let us consider y as a function of x, that is, y = f (x). This means any variation of x will affect y. Thus, we can say that y depends on x. It is represented as x → y. In other words, we can say there is an association exists between x and y. This means that x interacts with y. Now, consider the above example for gene expression data such that x and y are two genes. Thus, there exists an association if y = f (x), and gene x interacts with y. We call this interaction between gene x and y as gene–gene interaction.

Gene–gene interaction consists of weights between the genes and these weights help to decide the strength of that interaction. A gene interaction in normal sample will never be same with the diseased one. In the CRC sample either the interaction presents in the normal would never exist or a new interaction may develop due to mutation. Degree/weight of the interactions present in CRC samples represent degree of dependency between a pair of genes in a GRN. This will help the researchers to identify the real cause behind the CRC to take place. Similarly identifying the regulatory genes in a GRN will also be a great help as far as CRC is concerned. That's the reason why analysing gene – gene interaction, identifying their dependency and then finding the regulatory genes of CRC data set is really a challenge. Following section discusses about the proposed method in detail.

### 3.1 Initialization

Let's consider a microarray data set X of m genes, g_1_, g_2_, …, g_m_, each of which have n dimensions, representing samples. RBM is used here to extract the feature from one gene to identify the similarity with extracted feature of another gene. The proposed model first takes the input from two genes concurrently; say Gene g_1_ and Gene g_2_, and then trying to establish the interaction between g_1_ and g_2_ and moving next with g_1_ and g_3_ and so on. Gene g_1_ uses an RBM model to interact with Gene g_2_ which also uses another RBM. The Model uses a simple one‐layer Artificial Neural Network (ANN) having only one input and one output layer. When two RBMs interact with each other it uses a bridge to select the common output features from both sides of the model.

**FIGURE 2:**
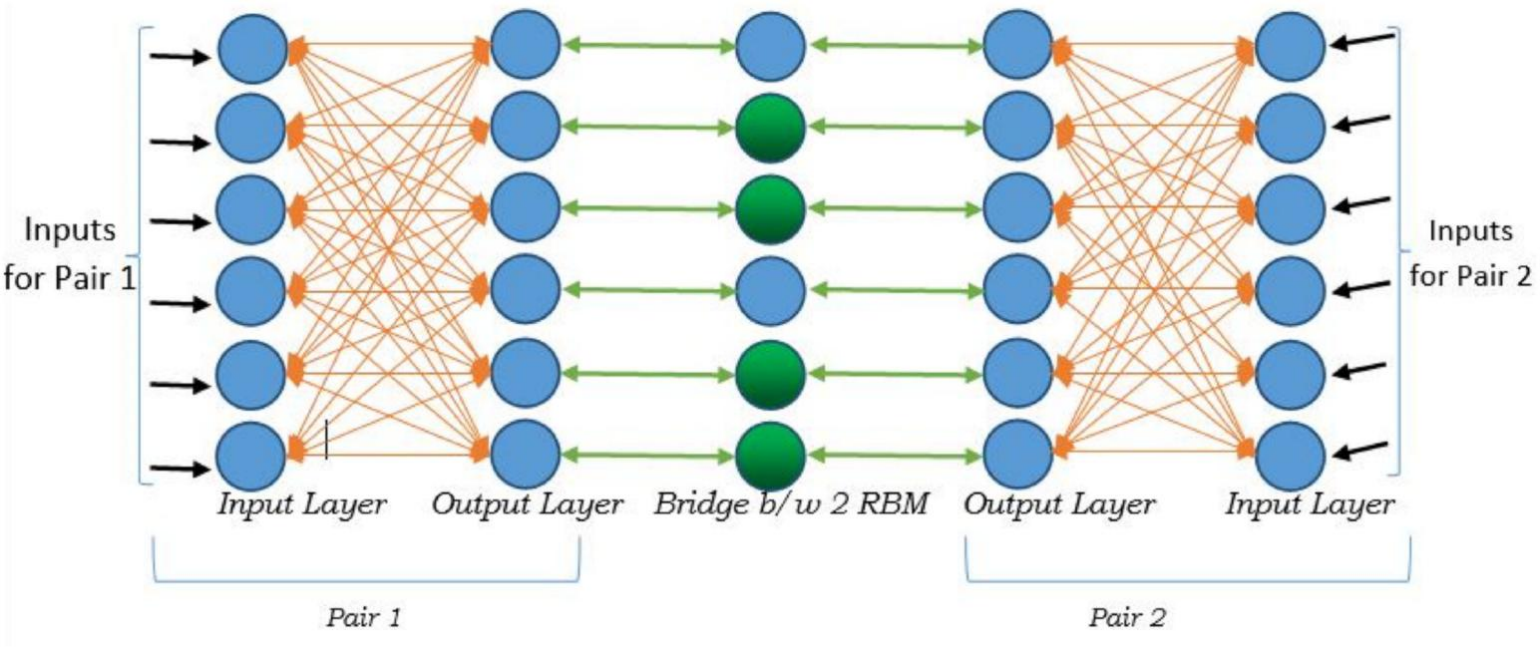
Restricted Boltzmann machine architecture

The architecture can be shown in Figure 2 as follows:

At the first iteration, the genes do not share information with each other using the bridge shown in Figure 2. They first learn the features of themselves by construction and reconstruction techniques. After one or more iterations g_1_ shares the information to g_2_ through the bridge for the reconstruction of the actual input of g_2_ and similarly g_2_ shares to g_1_ and try to reconstruct the actual input of g_1_. This continues until satisfactory minimum error of reconstruction of self inputs using the others is achieved. We choose this minimum error to be as low as 0.01. Any pair of gene interaction having error more than 0.01 is ignored.

Weights between input and hidden layer in the forward propagation are represented as w_1nxn_ and in the backward propagation are represented as w_2nxn_. These weight vectors are initialized as follows:

(2) $$w_{1\mathrm{nxn}}=w_{2\mathrm{nxn}}=\left[-1,\frac{1}{\sqrt{\mathrm{number}‐\mathrm{of}‐\mathrm{input}‐\mathrm{units}}}\right]$$

### 3.2 Pre‐processing stage

Given microarray dataset is normalized between 0 & 1 with the help of MAXMIN normalization using the following three steps:

(3) $$\mathrm{maxmin}=\max\left(g_{1}\left[1..n\right]\right)-\min\left(g_{1}\left[1..n\right]\right)$$

(4) $$g_{1}\left[1..n\right]=g_{1}\left[1..n\right]-\min\left(g_{1}\left[1..n\right]\right)$$

(5) $$g_{1}\left[1..n\right]=\frac{g_{1}\left[1..n\right]}{\mathrm{maxmin}}$$

Let's consider the following microarray dataset as an example containing five rows and six columns, as shown in Table 1.

**TABLE 1:** Sample microarray gene expression data set

	Sample 1	Sample 2	Sample 3	Sample 4	Sample 5	Sample 6
Gene 1	5.27055	5.07397	5.20949	5.25106	5.38327	5.13528
Gene 2	7.41519	7.11951	8.54414	7.73497	7.70082	8.04083
Gene 3	5.04245	5.01335	5.15868	4.58296	5.1552	4.58029
Gene 4	5.8967	5.82747	6.10051	5.71561	6.25209	5.9603
Gene 5	4.68734	4.62737	4.71932	4.77644	4.63229	4.29907

After applying MAXMIN normalization method on the above dataset we have the following normalized dataset, as shown in Table 2.

**TABLE 2:** Normalized version of Table 1

	Sample 1	Sample 2	Sample 3	Sample 4	Sample 5	Sample 6
Gene 1	0.6355	0	0.4381	0.5725	1	0.1982
Gene 2	0.2075	0	1	0.4320	0.4080	0.6467
Gene 3	0.7990	0.7487	1	0.0046	0.9939	0
Gene 4	0.3375	0.2085	0.7174	0	1	0.4561
Gene 5	0.8133	0.6877	0.8803	1	0.6980	0

### 3.3 Working procedure

As the data of the first gene g_1_ is given as input, forward propagation method calculates each output node using weights and bias. Once all the computations at the output nodes are done, then sigmoid activation function is used to nodes are done, then sigmoid activation function is used to calculate the activation probabilities between 0 and 1 for each of the output node at the output layer. At the same time similar kind of operations is performed for g_2_ from the other direction. Steps in detail are as follows:

Forward Propagation for g_1_

forward Hidden Node,

(6) $$fHg_{1}\left[1..n\right]=\sum_{i=1}^{n}b_{i}+g_{1}.w_{1}$$

forward Hidden activation function,

(7) $$fHg_{1}\text{act}\left[1..n\right]=\text{sigmoid}\left(fHg_{1}\right)$$

forward Hidden states,

(8) $$fHg_{1}\text{act}\left[1..n\right]=fHg_{1}\text{act}>\text{random\_state}$$

Forward Propagation for g_2_

forward Hidden Node,

(9) $$fHg_{2}\left[1..n\right]=\sum_{i=1}^{n}b_{i}+g_{2}.w_{2}$$

forward Hidden activation function,

(10) $$fHg_{2}\text{act}\left[1..n\right]=\text{sigmoid}\left(fHg_{2}\right)$$

forward Hidden states,

(11) $$fHg_{2}\text{act}\left[1..n\right]=fHg_{2}\text{act}>\text{random\_state}$$

Once forward propagation is done, the back propagation comes into play. The activation probabilities are used as new inputs and the last used weights and bias are considered to re‐construct the actual inputs using sigmoid function. The actual normalized inputs have been re‐constructed (r_i_) using back propagation method. Because the weights of the RBM are randomly initialized, the difference between the reconstructions and the original input (O_i_) is often large. It can be thought like a reconstruction error is then back propagated against the RBM's weights, again and again, in an iterative learning process until minimum error is reached.

Error is calculated as follows:

(12) $$\sum_{i}{\left(O_{i}-r_{i}\right)}^{2}$$

Detailed steps of backward propagation are shown as below:

Backward propagation for g_1_

if epoch = 1:

for backward visible node,

(13) $$bVg_{1}\left[1..n\right]=\sum_{i=1}^{n}b_{i}+fHg_{1}.w_{1}$$

else

(14) $$bVg_{1}\left[1..n\right]=\sum_{i=1}^{n}b_{i}+fHg_{2}.w_{1}$$

backward visible activation function,

(15) $$bVg_{1}\text{act}\left[1..n\right]=\text{sigmoid}\left(bVg_{1}\right)$$

backward hidden node,

(16) $$bHg_{1}\left[1..n\right]=\sum_{i=1}^{n}b_{i}+bVg_{1}\mathrm{act}.w_{1}$$

backward hidden activation function,

(17) $$bHg_{1}\text{act}\left[1..n\right]=\text{sigmoid}\left(bHg_{1}\right)$$

Backward propagation for g_2_

if epoch = 1:

for backward visible node,

(18) $$bVg_{2}\left[1..n\right]=\sum_{i=1}^{n}b_{i}+fHg_{2}.w_{2}$$

else

(19) $$bHg_{2}\left[1..n\right]=\sum_{i=1}^{n}b_{i}+fHg_{1}.w_{2}$$

backward visible activation function,

(20) $$bVg_{2}\text{act}\left[1..n\right]=\text{sigmoid}\left(bVg_{2}\right)$$

backward hidden node,

(21) $$bHg_{2}\left[1..n\right]=\sum_{i=1}^{n}b_{i}+bVg_{2}\mathrm{act}.w_{2}$$

backward hidden activation function,

(22) $$bHg_{2}\text{act}\left[1..n\right]=\text{sigmoid}\left(bHg_{2}\right)$$

On its forward pass, an RBM uses inputs to make predictions about node activations, or the probability of output given a weighted x: p (a| x; w), whereas on its backward pass, when activations are fed in and reconstructions, or guesses about the original data, an RBM is attempting to estimate the probability of inputs x given activations a, which are weighted with the same coefficients as those used on the forward pass. This second phase can be expressed as p (x| a; w).

To measure the distance between its estimated probability distribution and the ground‐truth distribution of the input, RBMs uses ‘Kullback–Leibler Divergence’. In our proposed method it is called as probability association. The actual input probability association for g_1_ is calculated using actual inputs and its activation probabilities generated at the output layer (g_1__fAssociation). The re‐constructed inputs are again passed through all the layer using forward propagation till it reaches the output layer using sigmoid activation function as activated hidden probabilities. Now, the reconstructed input is used to calculate re‐constructed probability association (g_1__bAssociation) with its activated hidden probability generated at the output layer.

Forward Association for g_1_ & g_2_

(23) $$g_{1}\_f\text{Association}=g_{1}\times fHg_{1}\text{act}$$

(24) $$g_{2}\_f\text{Association}=g_{2}\times fHg_{2}\text{act}$$

Backward Association for g_1_ & g_2_

(25) $$g_{1}\_b\text{Association}=bVg_{1}\mathrm{act}\times bHg_{1}\text{act}$$

(26) $$g_{2}\_b\text{Association}=bVg_{2}\mathrm{act}\times bHg_{2}\text{act}$$

Once this phase is finished, error is calculated for both g_1_ and g_2_ as follows:

(27) $$g_{1}\mathrm{error}=\sum_{i=1}^{n}{\left(g_{1}\left[i\right]-bVg_{1}\mathrm{act}\left[i\right]\right)}^{2}$$

(28) $$g_{2}\mathrm{error}=\sum_{i=1}^{n}{\left(g_{2}\left[i\right]-bVbg_{2}\mathrm{act}\left[i\right]\right)}^{2}$$

After that, weights are updated as per the following rules:

(29) $$w_{1}+=\text{learning\_rate}\times \frac{\left(g_{1}\_f\text{Association}-g_{1}\_b\text{Association}\right)}{\text{number}‐\text{of}‐\text{input}‐\text{units}}$$

(30) $$w_{2}+=\text{learning\_rate}\times \frac{\left(g_{2}\_f\text{Association}-g_{2}\_b\text{Association}\right)}{\text{number}‐\text{of}‐\text{input}‐\text{units}}$$

For the first iteration only the data of g_1_ are re‐constructed using its sigmoid activation probabilities generated from its actual inputs at the output layer and similarly for g_2_ data. This enables the g_1_/g_2_ to get knowledged of its own feature before getting the others. From the second iteration onwards the sigmoid activation probabilities of g_2_ are passed to g_1_ for re‐construction and g_1_ sigmoid activation probabilities are passed to g_2_ for re‐construction. Once the re‐construction is done the squared error and association are calculated to minimize the error and update the weights respectively. The number of iterations is continued till the model minimizes the error for the particular pair, here it is g_1_ and g_2_ or till the number of epochs.

At the last iteration, the input data of g_1_ are passed through all the layer and sigmoid activation probabilities are calculated where a random threshold is kept to activate the output node for g_1_. Similarly, for g_2_ data, same random threshold is kept to activate the output node at the last layer. Now each of the activated output node from g_1_ and g_2_ are compared. The matched activated nodes for the same level of nodes only, the activation probabilities are considered. If the node of g_1_ is activated at level 1 and at the same level g_2_ node is not activated, then the activation probability would not be considered for neither of the genes for that level of the output layer. When all the matched activated nodes are identified then mean of the activation probabilities for g_1_ and g_2_ is calculated.

Let's assume that w1 and w2 are the weights of g_1_ to g_2_ pair and g_2_ to g_1_ pair, respectively. No threshold has been considered to identify the strength between the gene pair rather low difference of mean value of *w*_1_ and *w*_2_ (as low as 0.01) was observed. If the difference of *w*_1_–*w*_2_ < 0.01 is satisfied for a particular gene pair then only it is accepted as valid gene–gene interaction, otherwise the interaction was not considered. Detailed steps to form a GRN are shown below:

The similarity has been measured by number of hidden states get activated between g_1_ and g_2_ and position where they are same with boolean value True.

g_1_hiddenactivestates[1..n] = g_1_hiddenactivationfunction[(g_1_hiddenstates == True) and (g_1_hiddenstates == g_2_hiddenstates)]
g_2_hiddenactivestates[1..n] = g_2_hiddenactivationfunction[(g_2_hiddenstates == True) and (g_1_hiddenstates == g_2_hiddenstates)]
#average of g_1_ and g_2_
g_1_weight = mean(g_1_hiddenactivestates[1..n])
g_2_weight = mean(g_2_hiddenactivestates[1..n])
if(abs(g_1_weight–g_2_weight) > 0.01)
break
Iteration end with the max number of epochs

Interactions are considered if (abs (g_1_weight–g_2_weight) <*θ*), where the value of the threshold (*θ*) is ranging from 0.004 to 0.016 and the direction of those interactions is identified in the next part.

Let's explain the above process with the help of the following Table 3 which shows the weight of the interactions between every pair of four genes after the specified number of epochs:

**TABLE 3:** Weight of the interactions after max epochs

	Gene 1	Gene 2	Gene 3	Gene 4
Gene 1	‐	0.765	0.772	0.698
Gene 2	0.779	‐	0.73	0.721
Gene 3	0.77	0.733	‐	0.7
Gene 4	0.6695	0.736	0.682	‐

Now, using the condition, if (abs (g_i_weight–g_j_weight) <=*θ*), where *i*≠j, 1 ≤ i, *j* ≤ 4, and threshold (*θ*) = 0.004, we got the following Table 4:

**TABLE 4:** Weight of the interactions after applying threshold

	Gene 1	Gene 2	Gene 3	Gene 4
Gene 1	‐	**0.765**	0.772	**0.698**
Gene 2	**0.779**	‐	0.73	**0.721**
Gene 3	0.77	0.733	‐	0.6843
Gene 4	**0.6695**	**0.736**	0.682	‐

Here, all the interactions marked in bold in Table 4 will be discarded, as the difference of the weights of those interactions > 0.004. So, we finally consider the likelihood of the interactions between Gene1–Gene3, Gene2–Gene3 and Gene3–Gene4.

The last phase in constructing the GRN is the identification of the regulatory genes. Gene regulation is a process used by any cell to control the production of specific gene products, like RNA, protein etc. A set of interactions between a pair of genes determine when and where specific genes are activated and the amount of protein or RNA product produced. To identify the regulatory genes in a GRN, NBC is applied on continuous valued attributes as follows:

(31) $$p\left(a_{k}|C_{i}\right)=g\left(a_{k},\mu_{ci},\sigma_{ci}\right)$$

where, the function g() is defined as follows:

(32) $$g\left(x,\mu ,\sigma \right)=\frac{1}{\sqrt{2\pi \sigma }}e^{-\frac{{\left(x-\mu \right)}^{2}}{2\sigma^{2}}}$$

Here, *µ*_Ci_ and σ_Ci_ are the mean & standard deviation, respectively, of the values of some attribute A_k_ for training tuples of class C_*i*_. A continuous valued attribute A_k_ is typically assumed to have a gaussian distribution and a_k_ ∈ A_k_ is a specific value of attribute A_k_, for which the probability to belong to the class C_*i*_ needs to be calculated. In our case, the target is to find which of the genes in a gene–gene pair has greater influence over the other. When it is found for all such pairs in the network then it can be clearly said that a gene controls which other genes, or is regulated by which other genes in the network. If we consider Gene1 – Gene2 pair, then we have two cases to consider. Either Gene1 regulates Gene2, that is Gene1 → Gene2, or vice‐versa, that is Gene2 → Gene1. That's why this is done in two phases. When Gene1 is regulated by Gene2, that is Gene1 → Gene2, we have found the mean and standard deviation of Gene2 and let's consider those values as *µ*_gene2_, σ_gene2_ respectively. Equations (31) and (32) are then applied on *µ*_gene2_, σ_gene2_ to find p(Gene1|Gene2). On the other hand, when Gene2 is regulated by Gene1, that is Gene2 → Gene1, we have found the mean and standard deviation of Gene1 and let's consider those values as *µ*_gene1_, σ_gene1_ respectively. Equations (31) and (32) are now applied on *µ*_gene1_, σ_gene1_ to find p(Gene2|Gene1). If p(Gene1|Gene2) > p(Gene2|Gene1), then Gene1 regulates Gene2, that is Gene1 → Gene2, otherwise Gene2 → Gene1.

What we observe from Table 4 that, there exist two strength values for each valid interaction, like between Gene1 and Gene3, two values are 0.772 and 0.73. Now, which of the values will dominate will be determined by NBC. Let's assume that the following Table 5 represent the sample gene expression values of the genes Gene1 and Gene3:

**TABLE 5:** Sample expression values of Gene 1 and Gene 3

Gene1	4.3078	4.5863	4.8488	4.7809	4.1483	3.8997	4.2633	4.2199	4.4515
Gene3	7.0182	6.6068	8.3893	8.1416	7.5879	6.7963	8.4705	7.2029	5.5523

Table 5 behaves here as training vector. Therefore, we have the following from Table 5 above:

*µ*_gene1_ = 4.389629
σ_gene1_ = 0.28986362569106
*µ*_gene3_ = 7.307352
σ_gene3_ = 0.89597257254372

So, using Equation (31) we have the following:

p(Gene1|Gene3) = 0.0032880123307121776
p(Gene3|Gene1) = 2.6393256102800502e−05

So, we can conclude that Gene1 is likely to be regulated by Gene3.

## 4 EXPERIMENTAL RESULTS

Whole experiments of the proposed approach are done on two CRC data sets of *Homo sapiens* available from NCBI repository [16]. One of them is GDS4382 and the other one is GDS1777.

### 4.1 Platform used

Platform: Jupyter Notebook—Python 3.5.2.

Libraries: sklearn, openpyxl, numpy, networkx and matplotlib.

### 4.2 Description of the data set used

GDS4382 is an analysis of paired CRC tumours and adjacent non‐cancerous tissues of human beings [16]. The series is published on 20 March 2012. The data set contains 34 samples, out of those 17 are control and the rest are diseased. All probe sets represented on this GeneChip Human Genome U133 Set are identically replicated on the Affymetrix Human Genome U133 Plus 2.0 Array.

GDS1777 is another analysis of colon tubular adenoma and carcinoma cells micro‐dissected from formalin‐fixed paraffin‐embedded sections of colon tubular adenomas containing focal adenocarcinomas of human beings [16]. This series is published on 22 December 2005. The data set contains eight samples. The platform on which the probe sets of this data set are represented is Human‐8K cDNA microarray.

### 4.3 Results

On the way to perform our experiments, we first rank the genes of GDS4382 data set by using Wilcoxon Rank Sum Test. Then we try to match the genes in the ranked set from NCBI Colorectal Biosystem. In this way we select top 50 genes that matches with NCBI. Among these top 50 genes we have found that 20 genes are common to GDS1777 data set and we have taken those 20 genes for our experiment. We further checked that if we take top 100 genes instead of top 50, then also there is no change in number of common genes found. We have divided these 20 genes into a sequence of 1–10 (first set of result) and 11–20 (second set of result) genes. First, we have used GDS4382 data set to observe the change of interaction between Normal and Diseased samples of human CRC. Here, normal genes refer to the healthy genes that exhibit proper/normal cell growth, whereas diseased genes are identified as the mutant genotypes responsible for an inherited genetic disorder or responsible for abnormal cell growth.

**FIGURE 3:**
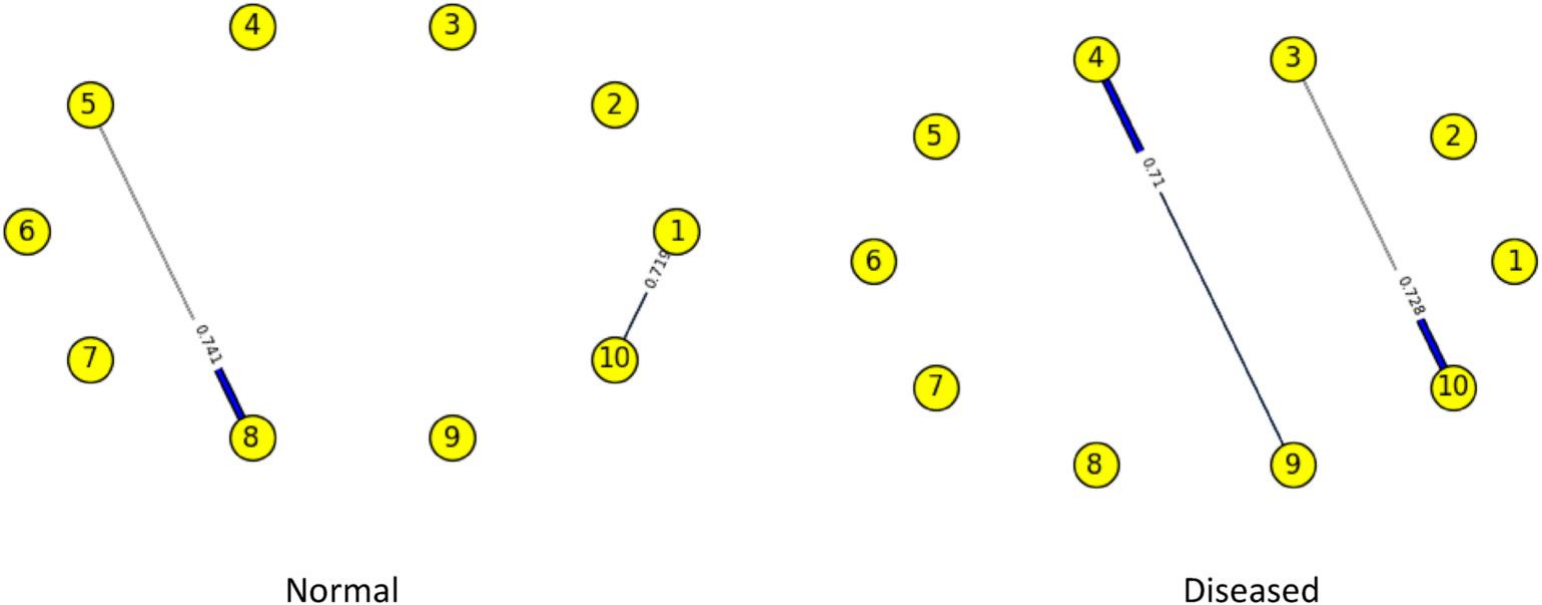
Gene–Gene interactions present in Normal and Diseased samples of first set of 10 genes of GDS4382

**FIGURE 4:**
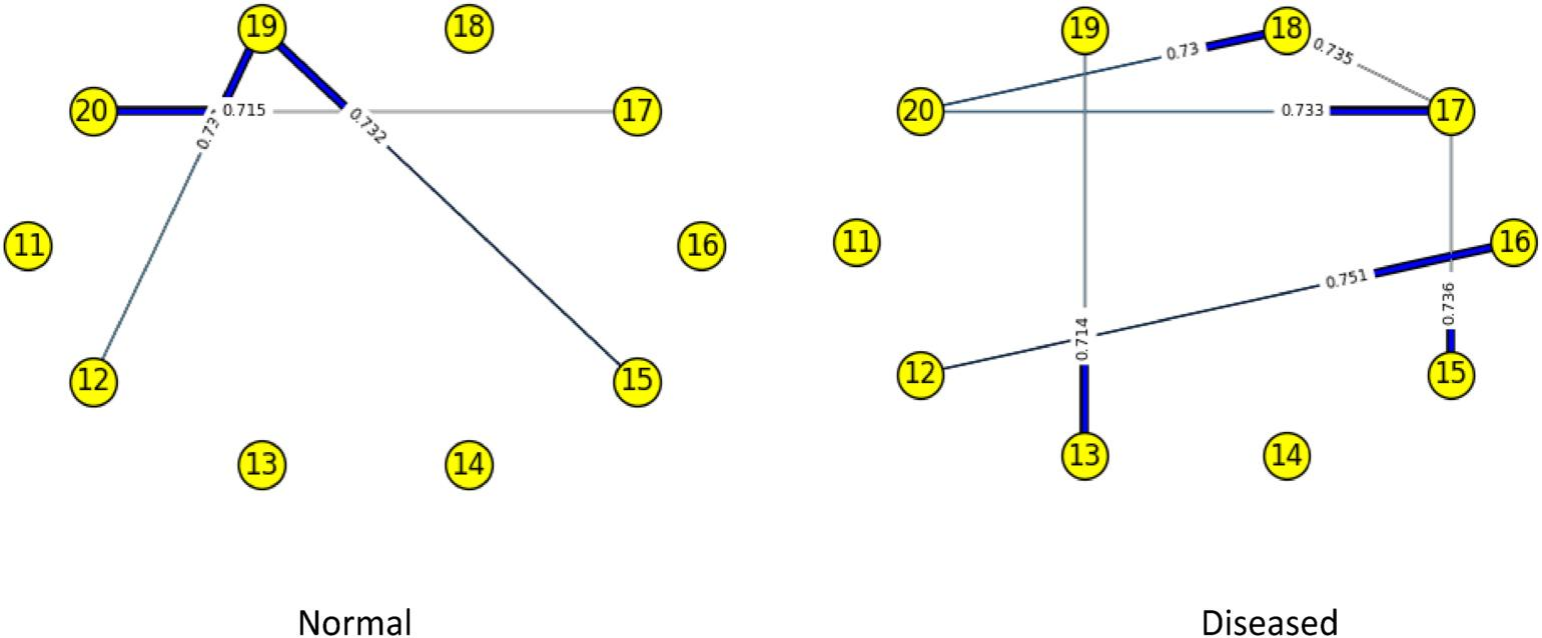
Gene–Gene interactions present in Normal and Diseased samples of next set of 10 genes of GDS4382

**FIGURE 5:**
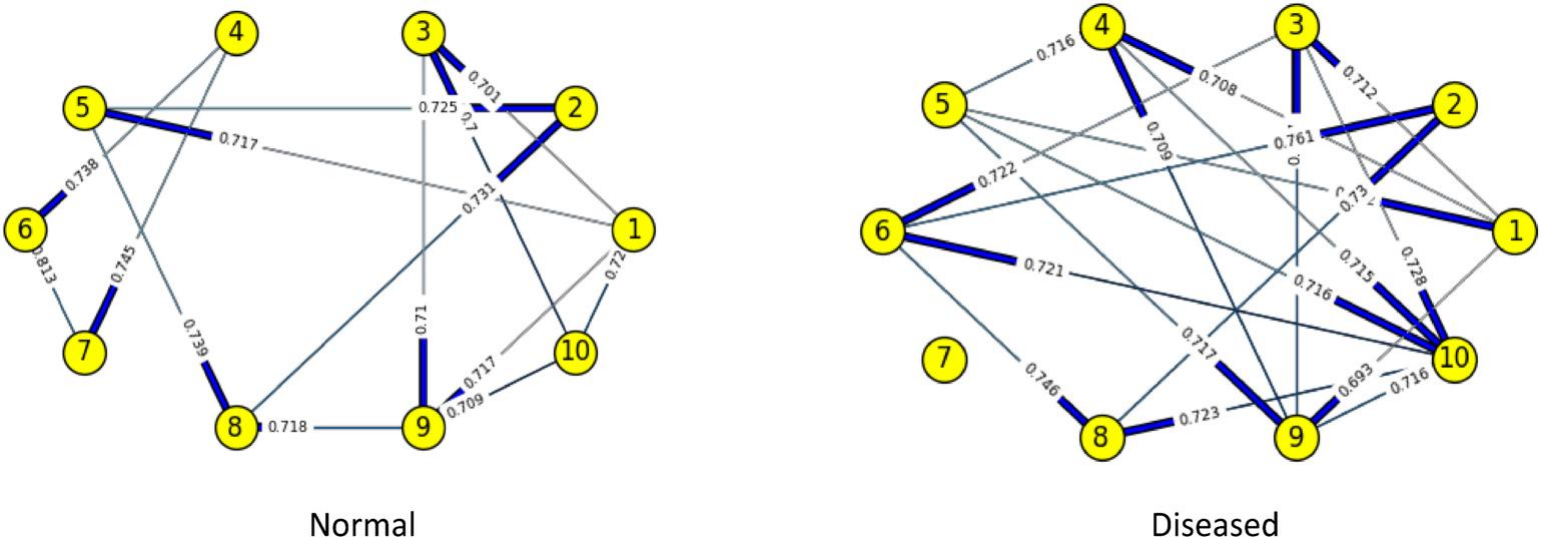
Gene–Gene interactions present in Normal and Diseased samples of first set of 10 genes of GDS4382

**FIGURE 6:**
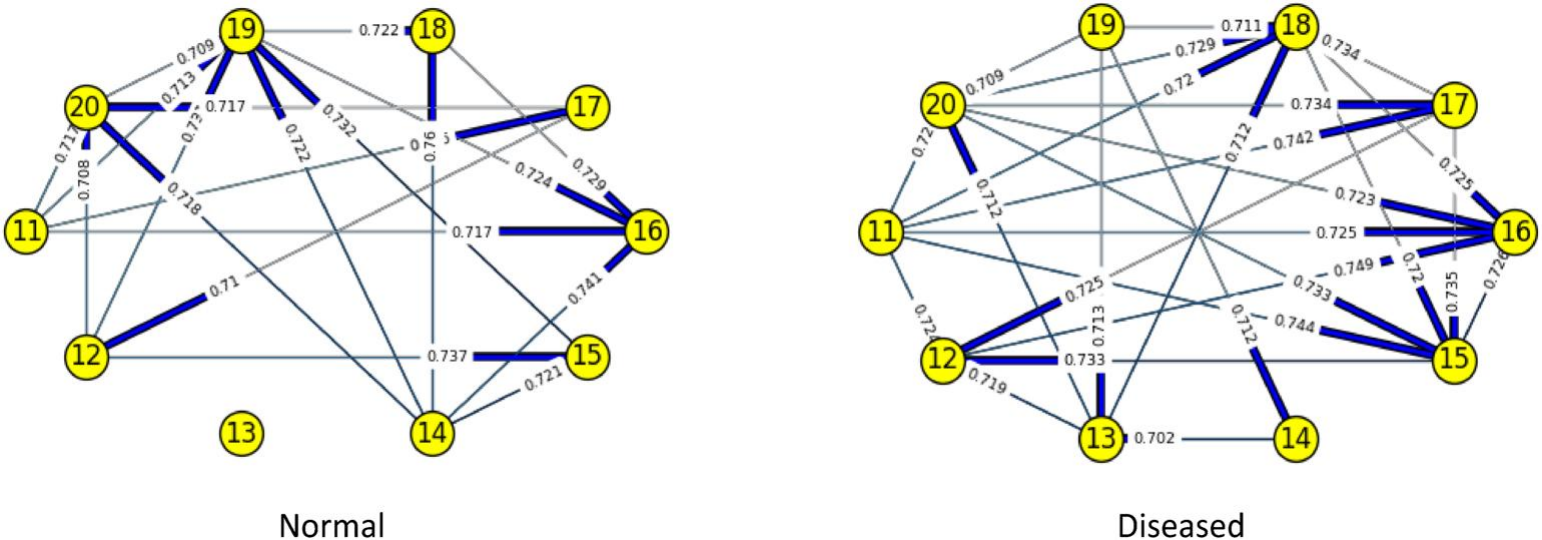
Gene–Gene interactions present in Normal and Diseased samples of next set of 10 genes of GDS4382

**FIGURE 7:**
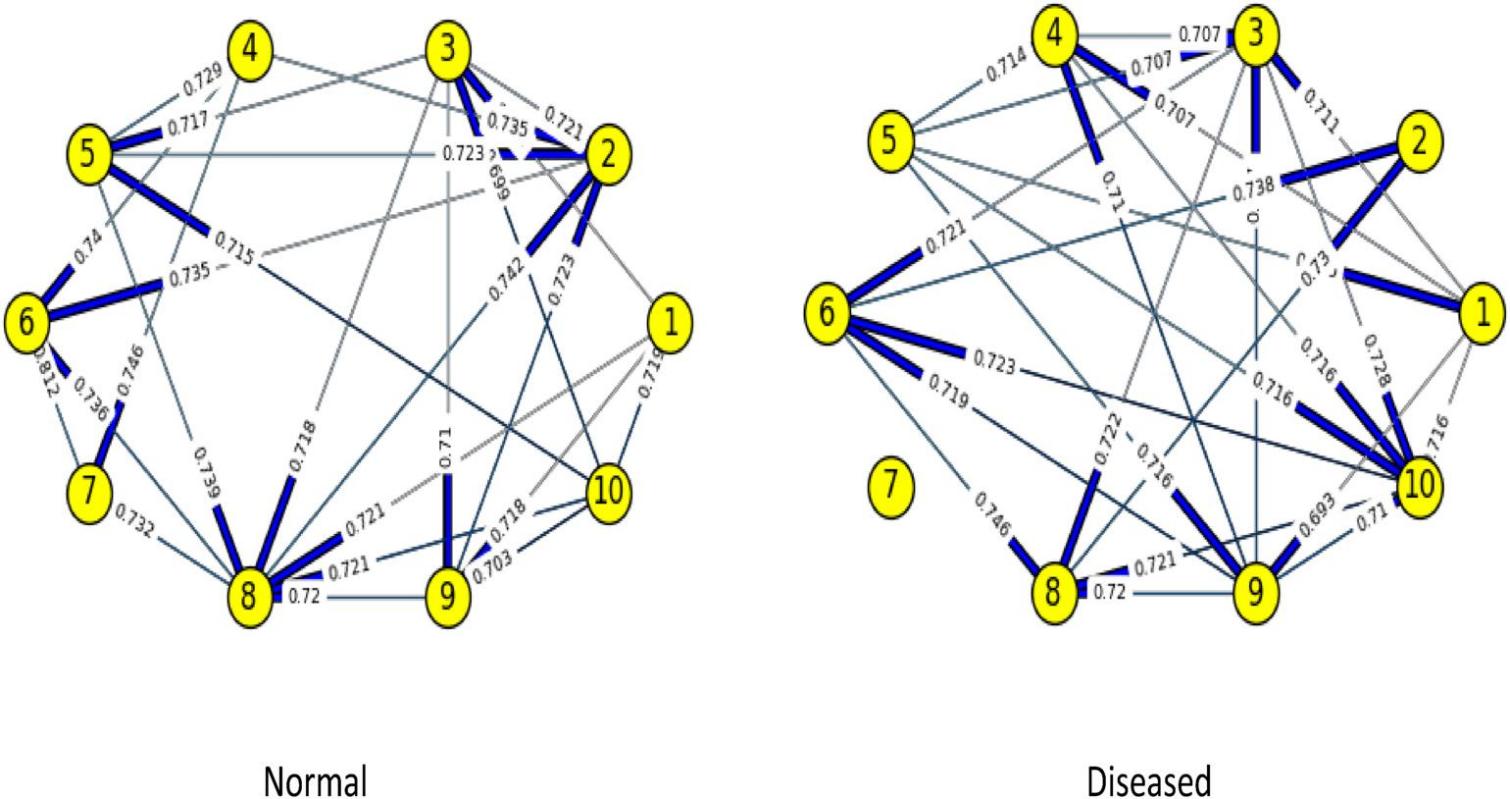
Gene–Gene interactions present in Normal and Diseased samples of first set of 10 genes of GDS4382

**FIGURE 8:**
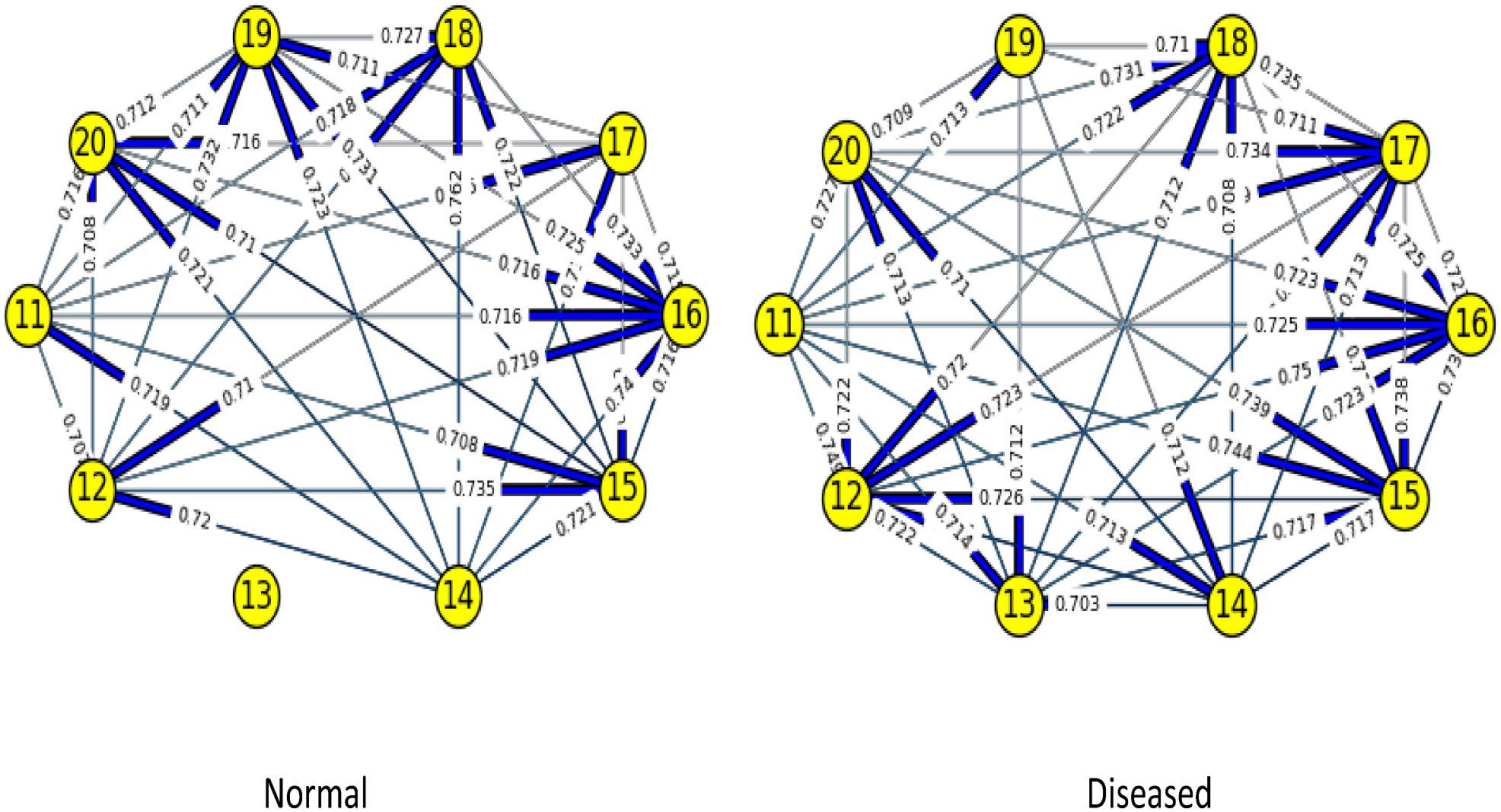
Gene–Gene interactions present in Normal and Diseased samples of next set of 10 genes of GDS4382

Figures 3, 4, 5, 6, 7, 8 below shows GRNs for Normal & Diseased samples of GDS4382, whereas Figures 10, 11, 12, 13, 14, 15 displays the same type of networks for Adenoma and Carcinoma samples of GDS1777 for a range of thresholds *θ*. Nodes in those figures represent the genes, for which the corresponding gene names are mentioned in the Table 3 below, and the directed edges represent the interactions identified by our experiments. If there is a directed edge from gene A to gene B in any GRN, that is A→B, that means gene B is regulated by gene A, in turn its biological significance is that the expression values of gene B is controlled by the expression value of gene A. The values present on the edges represent the strength of that interaction. The same gene sequence was taken and the experiment was repeated once again with GDS1777 (Adenoma and Carcinoma genes) data set. Adenoma genes are responsible for benign tumour and Carcinoma genes are responsible for abnormal cell growth that leads to cancer. This experiment will convey us about the presence or absence of those interactions in the diseased samples, as well as in the different phases of the diseased samples, like Adenoma and Carcinoma, that may be responsible for that particular cancer to take place. Following Figure 3 shows the interactions present between the first 10 genes of Normal samples and diseased samples of GDS4382 data set separately when *θ* = 0.006.

**FIGURE 9:**
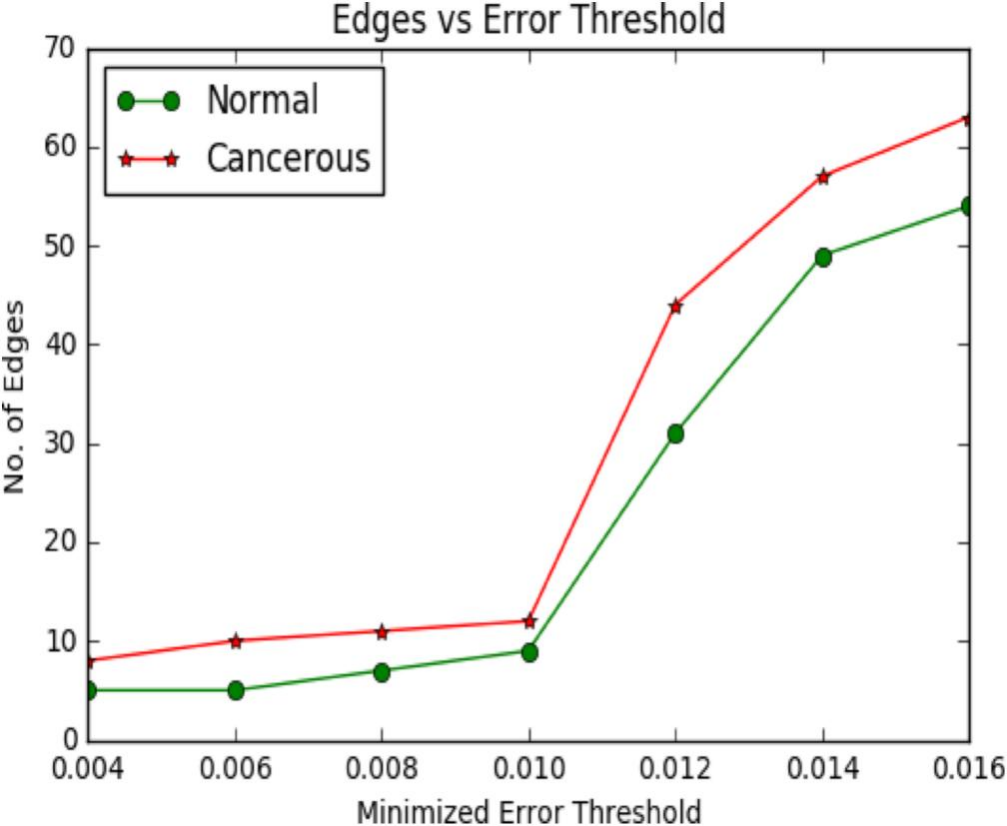
Plot showing difference in edges versus error threshold

**FIGURE 10:**
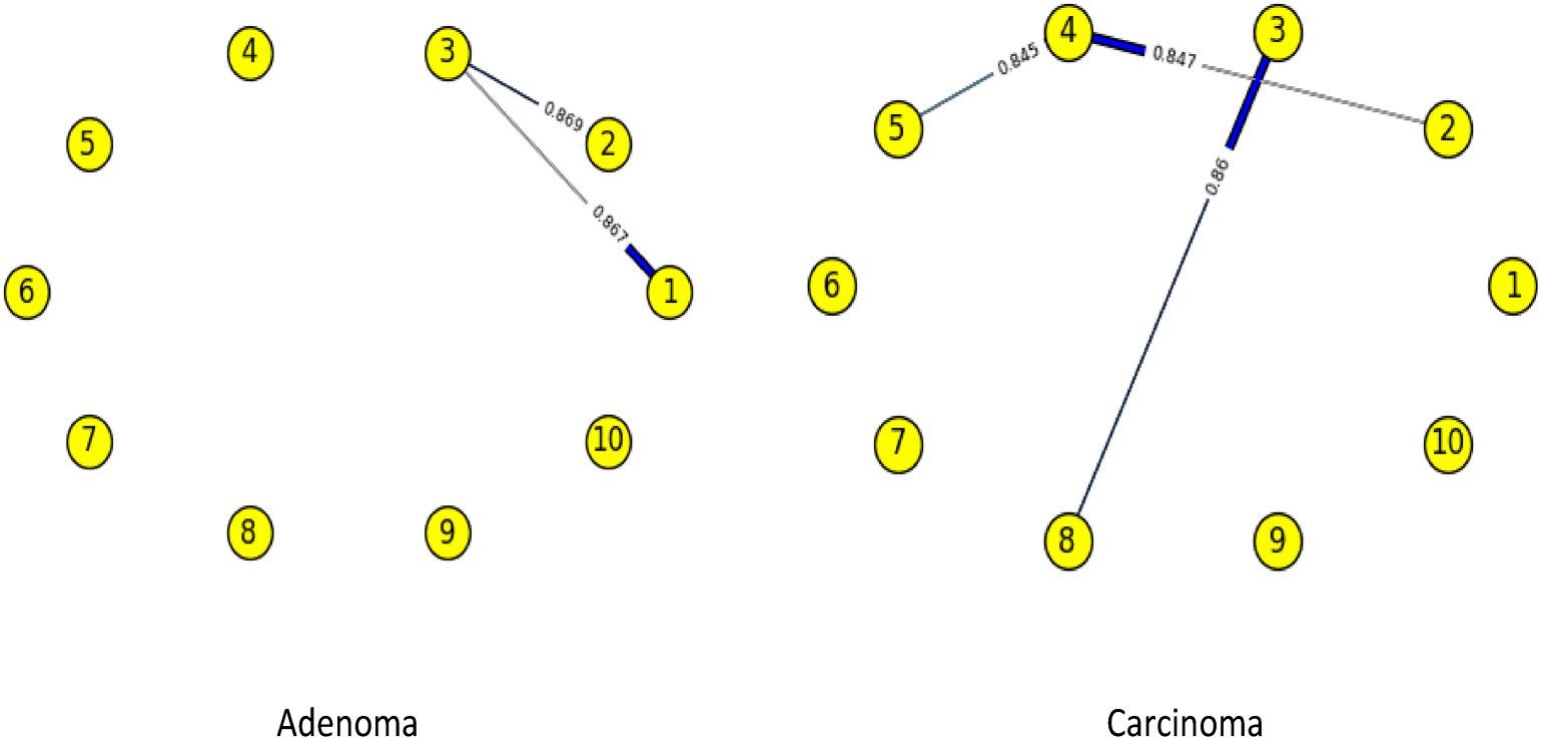
Gene–Gene interactions present in Adenoma & Carcinoma samples of first set of 10 genes of GDS1777

**FIGURE 11:**
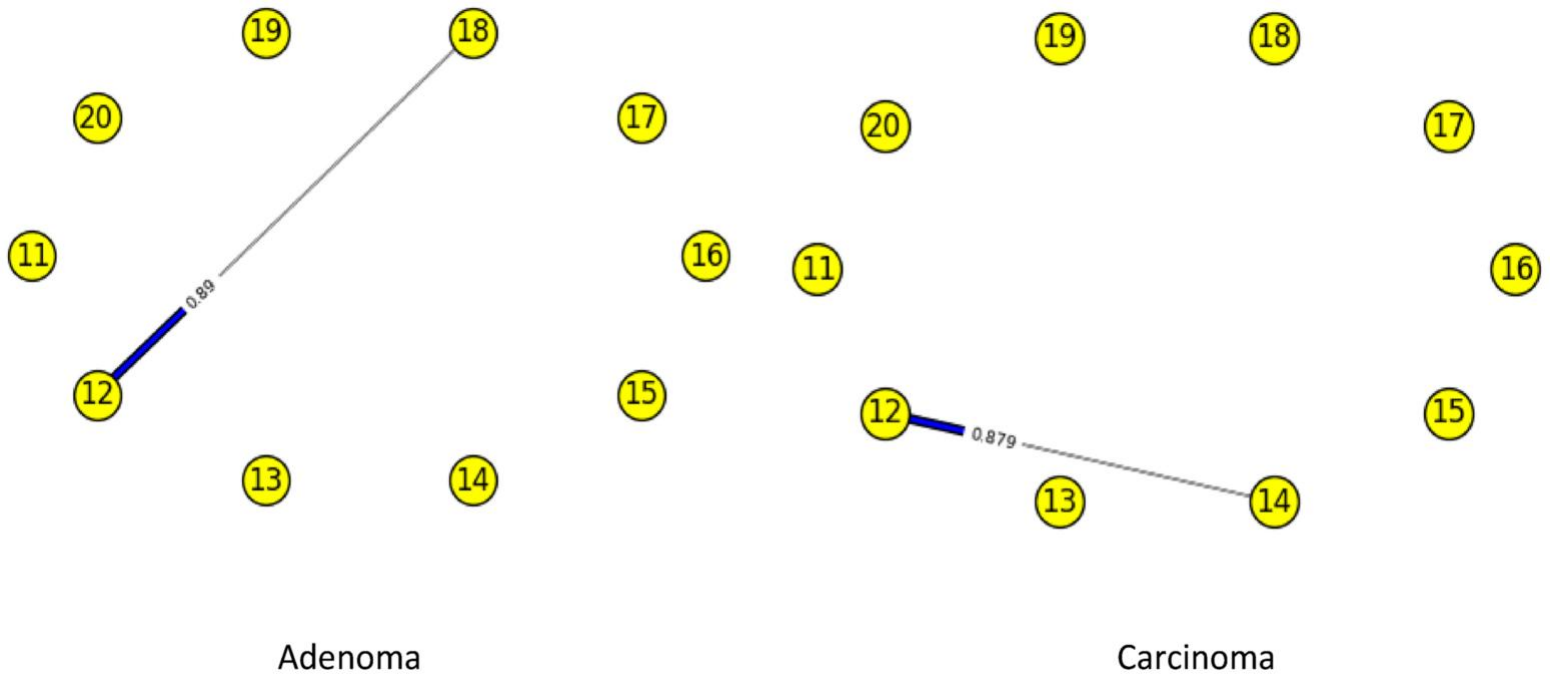
Gene–Gene interactions present in Adenoma & Carcinoma samples of next set of 10 genes of GDS1777

**FIGURE 12:**
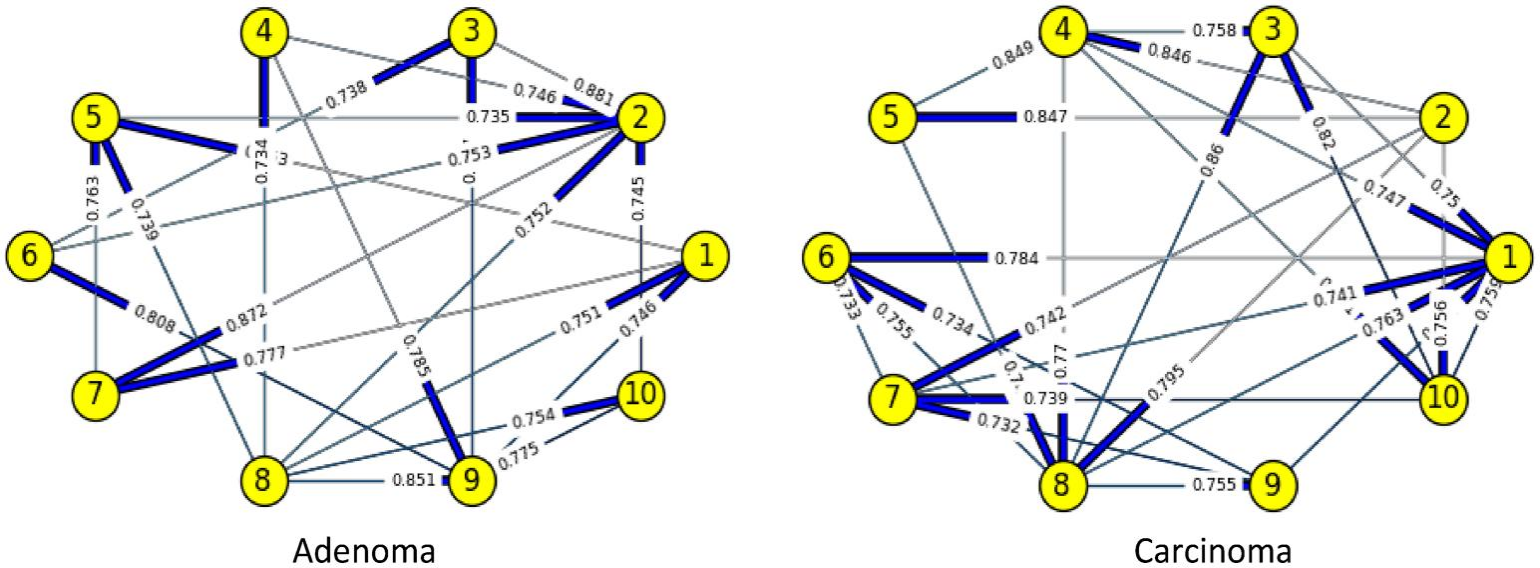
Gene–Gene interactions present in Adenoma & Carcinoma samples of first set of 10 genes of GDS1777

**FIGURE 13:**
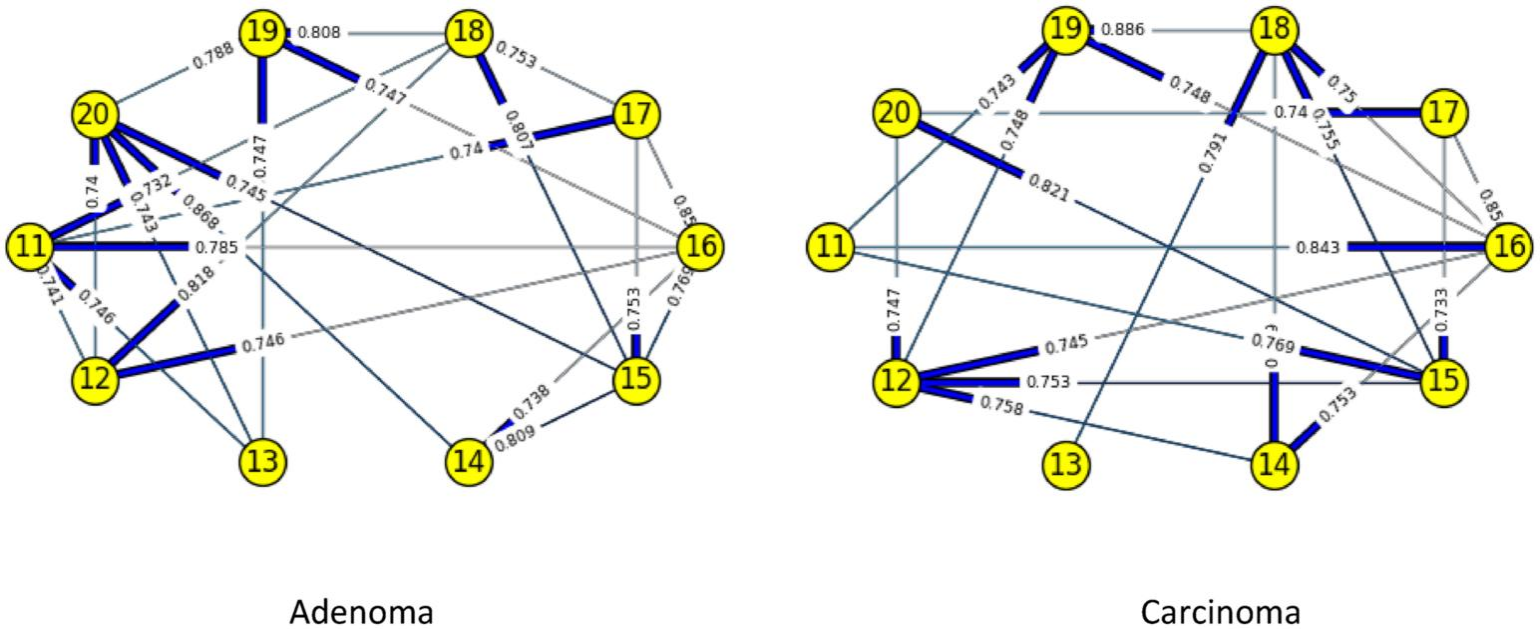
Gene–Gene interactions present in Adenoma & Carcinoma samples of next set of 10 genes of GDS1777

**FIGURE 14:**
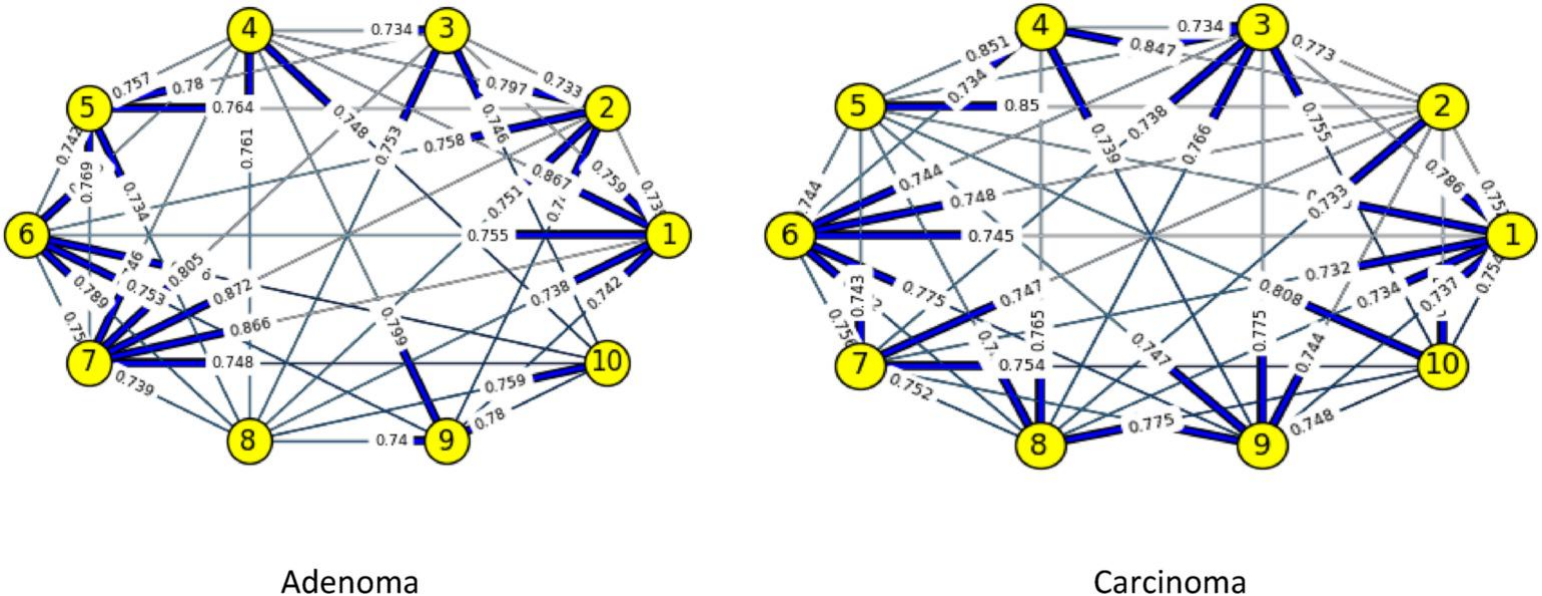
Gene–Gene interactions present in Adenoma & Carcinoma samples of first set of 10 genes of GDS1777

**FIGURE 15:**
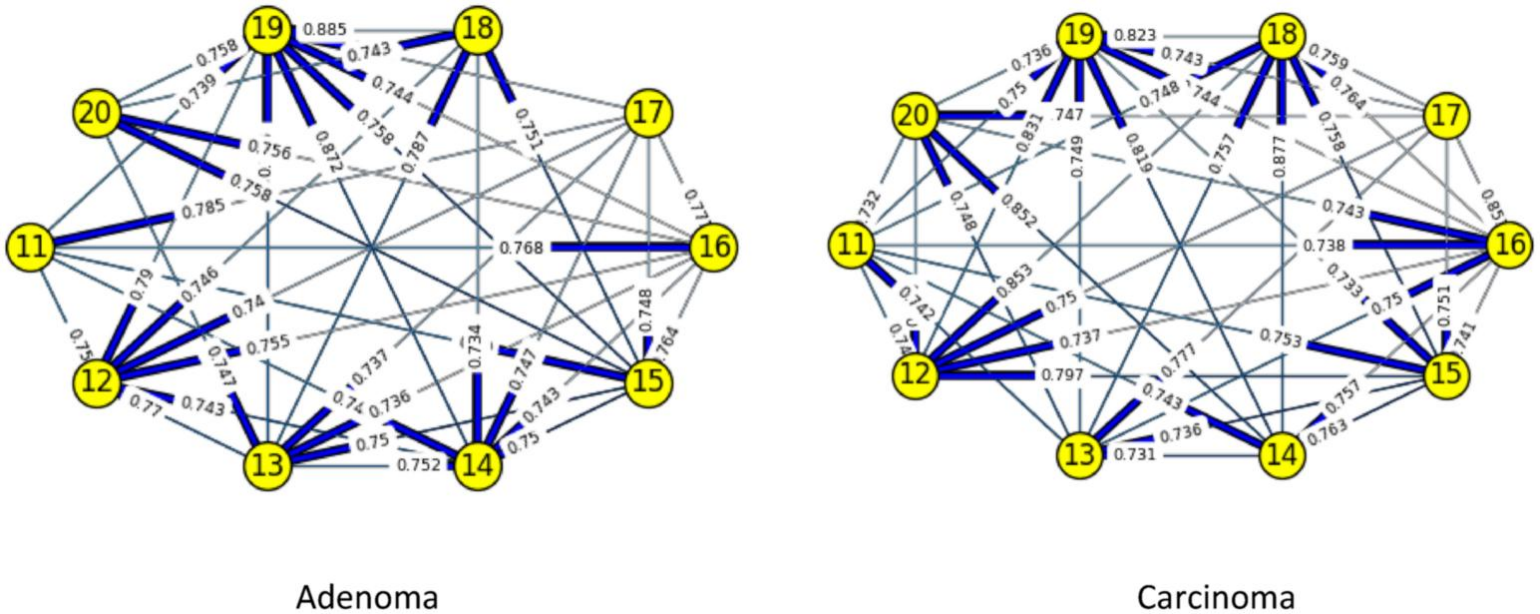
Gene–Gene interactions present in Adenoma & Carcinoma samples of next set of 10 genes of GDS1777

Following Figure 4 shows the interactions present between the next set of 10 genes of Normal samples and diseased samples of GDS4382 data set separately when *θ* = 0.006.

Following Figure 5 shows the interactions present between the first 10 genes of Normal samples and diseased samples of GDS4382 data set separately when *θ* = 0.012.

Following Figure 6 shows the interactions present between the next set of 10 genes of Normal samples and diseased samples of GDS4382 data set separately when *θ* = 0.012

Following Figure 7 shows the interactions present between the first 10 genes of Normal samples and diseased samples of GDS4382 data set separately when *θ* = 0.016.

Following Figure 8 shows the interactions present between the next set of 10 genes of Normal samples and diseased samples of GDS4382 data set separately when *θ* = 0.016.

Following plot of Figure 9 shows how the number of edges between the normal and diseased samples is changed with the variation of the threshold *θ*.

From the above plot, we observed that, with the increasing value of threshold, the no. of edges increases for both normal and cancer data. Further, we observed that the number of edges in the diseased network is always outnumbered the normal network and the difference between them increases with increase in the threshold value, that is, with the increase in the number of edges the difference between the diseased network and normal network increases.

Next experiment is carried on the same 20 genes of GDS1777, which shows the two stages of cancer, that is Adenoma and Carcinoma. Adenoma is a type of non‐cancerous tumour or benign that may affect various organs. Carcinoma is a type of cancer that starts in cells that make up the skin or the tissue lining organs, such as the liver or kidneys.

Following Figure 10 shows the interactions present between the first 10 genes of Adenoma and Carcinoma samples of GDS1777 data set separately when *θ* = 0.006.

Following Figure 11 shows the interactions present between the next set of 10 genes of Adenoma and Carcinoma samples of GDS1777 data set separately when *θ* = 0.006.

Following Figure 12 shows the interactions present between the first set of 10 genes of Adenoma and Carcinoma samples of GDS1777 data set separately when *θ* = 0.012.

Following Figure 13 shows the interactions present between the next set of 10 genes of Adenoma and Carcinoma samples of GDS1777 data set separately when *θ* = 0.012.

Following Figure 14 shows the interactions present between the first set of 10 genes of Adenoma and Carcinoma samples of GDS1777 data set separately when *θ* = 0.016.

Following Figure 15 shows the interactions present between the next set of 10 genes of Adenoma and Carcinoma samples of GDS1777 data set separately when *θ* = 0.016.

In order to validate the interactions as well as the direction of those interactions between a pair of genes in GRNs, we have checked whether those interactions are already reported at NCBI database or not. We have validated the likelihood of the interactions and the corresponding directions of those interactions through NCBI database [16] and also using some earlier investigations [17]. Here, we explain the validation of the interactions among the genes in brief for the better understanding of the readers. In most of the GRNs for Normal samples we have found that the gene AKT2 (AKT serine/threonine kinase2) regulates PIK3CD (phosphatidylinositol‐4,5‐bisphosphate 3‐kinase catalytic subunit delta) and this was also reported at NCBI [16]. Similar to this investigation, some of the other interactions can also be mentioned, like CCND1 (cyclin D1) is regulated by FOS (Fos proto‐oncogene), CASP3 (caspase 3) regulates CASP9 (caspase 9), SMAD3 (SMAD family member3) regulates SMAD2 (SMAD family member2), AXIN1 (axin1) regulates SMAD3 (SMAD family member3), FOS (Fos proto‐oncogene) controls SMAD3 (SMAD family member3). In this way, we have validated the result as well as the direction of the interaction among the genes through NCBI database.

**FIGURE 16:**
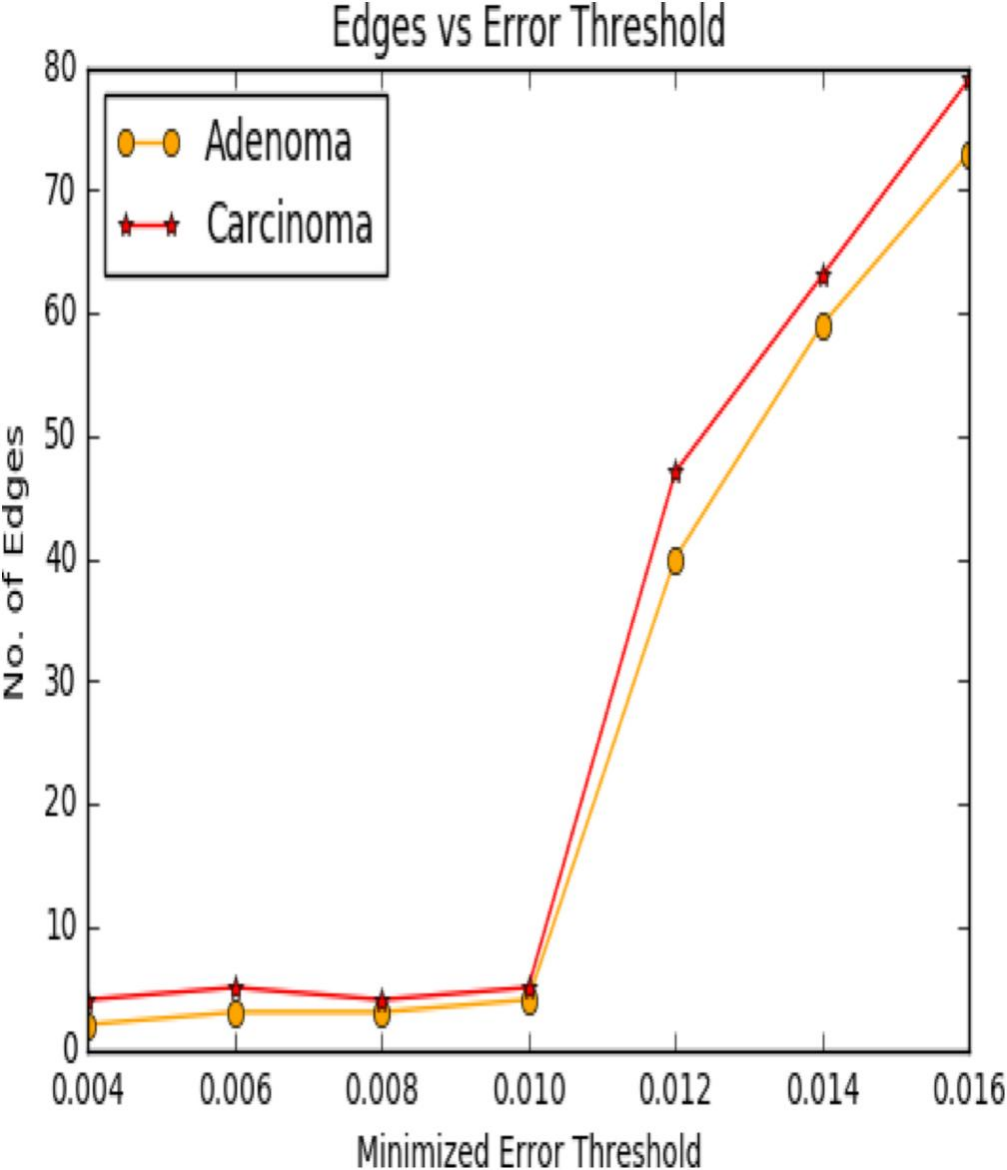
Plot showing difference in edges versus error threshold

Following plot of Figure 16 shows how the number of edges between the adenoma and carcinoma samples of these 20 genes is changed with the variation of the threshold *θ*.

Following Table 6 shows the mapping of the node numbers in the GRN to the gene names of those 20 genes used in the experiment for both GDS4382 and GDS1777 data sets.

**TABLE 6:** Node no.—Gene name mapping

GDS4382		GDS1777	
Node no.	Gene name	Node no.	Gene name
1	AKT2	1	AKT2
2	PAK4	2	SMAD2
3	PAK2	3	PIK3R1
4	SMAD2	4	PAK2
5	PIK3R1	5	PAK4
6	CCND1	6	CCND1
7	AXIN1	7	AXIN1
8	FOS	8	FIGF
9	SMAD3	9	SMAD3
10	PIK3CD	10	PIK3CD
11	TGFB2	11	TGFB2
12	ARAF	12	ARAF
13	MLH1	13	MLH1
14	PIK3CB	14	PIK3CB
15	TCF7L2	15	TCF7L2
16	CASP3	16	CASP3
17	CASP9	17	CASP9
18	APPL1	18	APPL1
19	MAPK10	19	MAPK10
20	TCF7	20	TCF7

We can compare our work with another work mentioned by Saha et al. [15], where correlation measures, relative entropy and rough set theory are used to find the interaction and the strength of the interaction between a pair of genes in a gene interaction network. Although the work is similar but there are some differences. First of all, the work by Saha et al. [15] is applied on GDS 4382 data set, that is, it only tries to find out the interactions between the normal and diseased samples of CRC data set. In our work, we use GDS4382 as well as GDS1777. That means apart from finding the interactions between normal and diseased samples, we are also analysing the different stages of CRC disease, that is, our work is also finding the interactions between adenoma and carcinoma samples. Cancer development signifies an abnormal growth of cells and depending on this growth the stages of a cancer is identified. As we know a cell cycle involves gene interaction and the changes of interaction between normal and diseased/adenoma and carcinoma samples can be due to mutation and other conditions. Thus, we also observed that the number of interactions between diseased genes are increased than the normal genes, whereas the number of interactions between carcinoma genes are more than the adenoma genes. Another difference lies in the working principle of both the works. Both the datasets that we have used in our RBM based model are time series data. That means if we take the snapshot of the data set in two different time instances, then we can have different gene interaction networks. In RBM based model the weights between the layers are initialized with random values, thus every time we run the proposed model, 10%–20% of the total interaction changes between the genes of normal and diseased samples though most of the interaction remains the same. Thus, it maintains the real‐life scenario. The above obversions were not visible in the work [15], because that work uses the correlation measures ranging from 0.15–0.8 [for positive] and (−0.4)–(−0.1) [for negative]. Since correlation is a statistical measurement the calculation does not involve any random values, and thus does not show any dynamic behaviour. That means this work does not reflect the actual scenario.

## 5 CONCLUSIONS AND FUTURE SCOPES

Objective of this work is to find the weight of the interaction, as well as the direction of the interaction present in normal & diseased samples and in adenoma and carcinoma samples of every pair of genes in a GRN of CRC data set. RBM, along with NBC is used over here to achieve this objective. We are actually looking for the likelihood of those interactions that are present in normal samples, but not present in diseased samples, or vice versa. We are also interested in finding the likelihood of those interactions present in adenoma samples, but not in carcinoma samples, or vice versa. This is because the presence/absence of those interactions may be responsible for the CRC to take place. As for example, from Figure 3 it is shown that, there is an interaction between genes 5 & 8 in normal samples, but that interaction is not present in the diseased samples. Whereas the interaction between genes 2 & 6 is present in diseased sample, but that was not present in the normal sample. The weight/degree of the interaction represents the strength of the interaction between the genes. It indicates how strong/weak an interaction is. It has seen that in a GRN, one gene regulator controls another, and so on. Gene regulation is very much significant for viruses, prokaryotes, eukaryotes etc. The reason is it increases the versatility and adaptability of an organism by allowing the cell to express protein when it is needed. The process that is followed here to find the gene regulation is very much similar to the working principle of NBC. That's why we have used it here. At this phase, it was observed that, mostly the new interactions are formed in the diseased genes that were not present in Normal genes and if the common interaction exists between normal and diseased genes then in most of the cases the direction gets reversed. The same gene sequences were taken and the experiment was repeated once again on GDS1777 (Adenoma and Carcinoma samples) data set. Adenoma genes are responsible for benign tumour and Carcinoma genes are responsible for abnormal cell growth that leads to cancer. We observed that interaction changes from Adenoma to Carcinoma in an unpredictable manner for the same set of genes. A mutation is a change that creates an abnormal protein or it may prevent a protein's formation. Mutation of genes will alter the gene–gene interaction or protein‐protein interaction which was observed in our experiment. What we observe here is that the interaction not only changes from Adenoma to Carcinoma but if common interaction exists then their direction also gets changed. The change of interaction between pairs of genes is more likely to be the part of gene mutation which is responsible for that particular disease.

In this work we have used one specific model of Deep Learning Neural network, that is, the RBM model to find the level of interaction between the genes. As a future scope we have a plan to use some other deep learning neural net models, like convolutional neural network models and so on for doing the same job.
